# Supplementary material for: Clinical Trial on the Safety and Tolerability of Personalized Cancer Vaccines Using Human Platelet Lysate-Induced Antigen-Presenting Cells
Source: Cancers (Basel). 2023 Jul 14;15(14):3627. doi: 10.3390/cancers15143627 (PMC10377585; doi:10.3390/cancers15143627)
Supplement: Supplementary file 1 [file cancers-15-03627-s001.zip › cancers-2446234-supplementary.pdf]

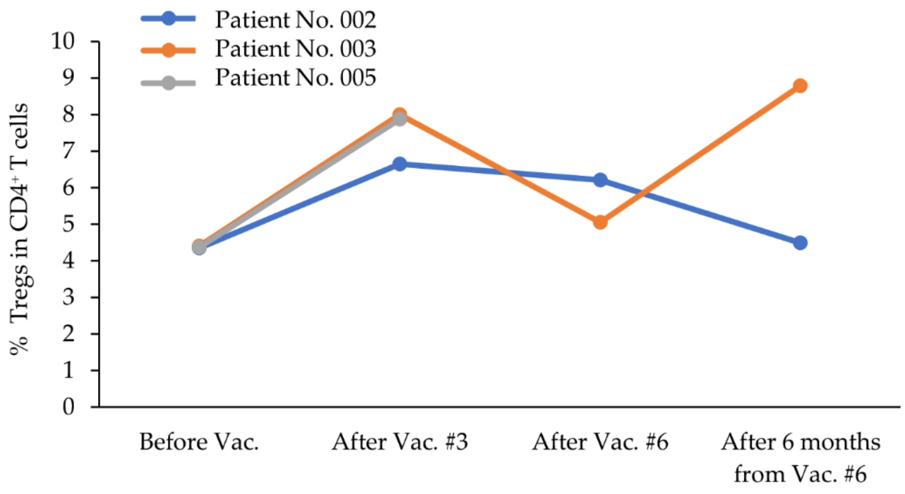

(a)

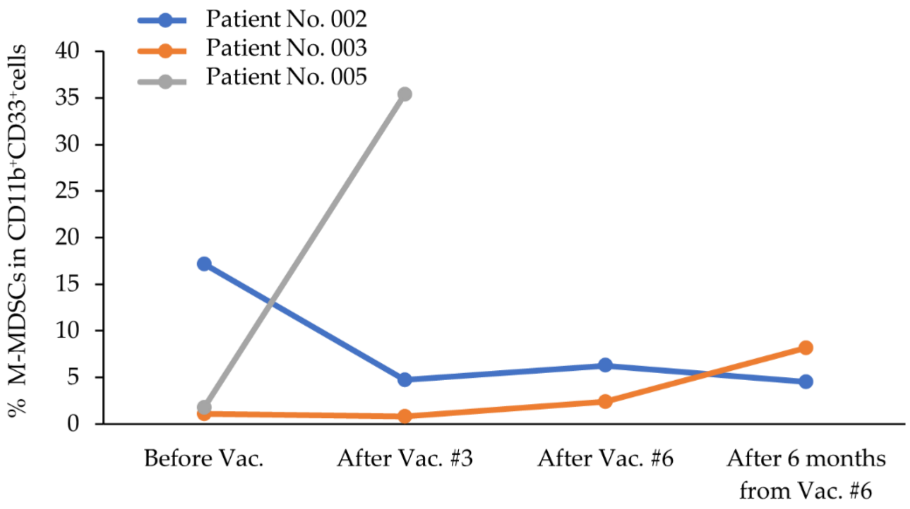

(b)

Figure S1. Tregs (**a**) and M-MDSCs (**b**) in patients treated with HPL-APC vaccines.

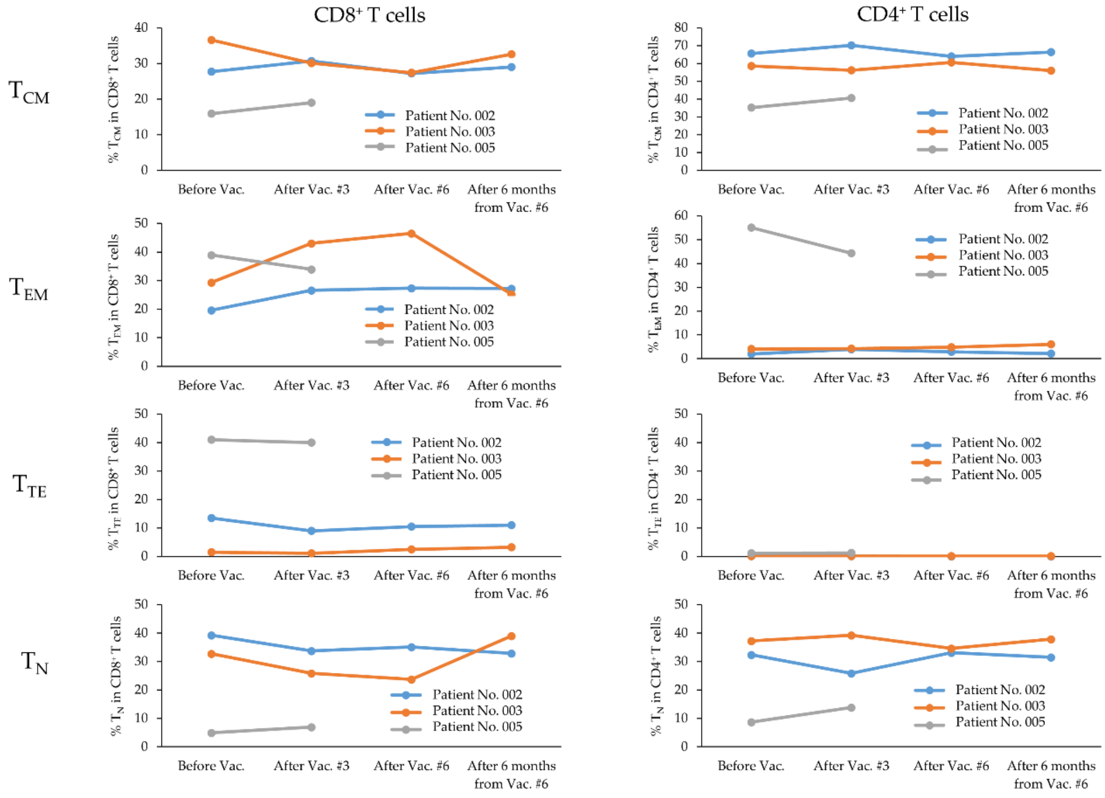

Figure S2. Memory T-cell subsets in patients treated with HPL-APC vaccines.

Table S1. Antibodies for HPL-APC.

Antibody	Fluorescence	Clone	Source
CD40	FITC	5C3	Thermo Fisher Scientific
CD80	FITC	L307.4	BD Biosciences
CD86	PE	IT2.2	Thermo Fisher Scientific
HLA-ABC	FITC	G46-2.6	BD Biosciences
HLA-DR	PE	LN3	Thermo Fisher Scientific

Table S2. Prediction of HLA-matched HIV peptides.

Patient No.	HLA type	Sequence	Peptide name	Affinity to HLA (nM)
2	HLA-A*24:02	RYLRDQQLL	HIV-HLA-A*24:02	36
	HLA-A*33:03	MVHQAISPR	HIV-HLA-A*33:03	10
3	HLA-A*11:01	HQAAMQMLK	HIV-HLA-A*11:01	23
	HLA-A*31:01	IMMQRGNFR	HIV-HLA-A*31:01	8
5	HLA-A*02:06	VLAEAMSQV	HIV-HLA-A*02:06	6
2	HLA-DRB1*07:01	KIVRMYSPTSILDIR	HIV-HLA-DRB1	28
3	HLA-DRB1*04:01			69
5	HLA-DRB1*01:01			18
2, 3, 5	HLA-DRB1*09:01			62

Table S3. Antibodies for Tregs.

Antibody	Fluorescence	Clone	Source
CD3	FITC	SK7	BD Biosciences
CD4	APC-Cy7	SK3	BioLegend
CD25	PE	2A3	BD Biosciences
CD45	BV510	2D1	BioLegend
CD127	BV421	A019D5	BioLegend

Table S4. Antibodies for M-MDSCs

Antibody	Fluorescence	Clone	Source
CD11b	APC-Cy7	ICRF44	BioLegend
CD14	FITC	61D3	Invitrogen
CD33	PE-Cy7	WM53	BioLegend
CD45	BV510	2D1	BioLegend
HLA-DR	PE	LN3	Invitrogen

Table S5. Antibodies for memory T-cell subsets.

Antibody	Fluorescence	Clone	Source
CD3	FITC	SK7	BD Biosciences
CD4	BV421	SK3	BioLegend
CD8	BV421	SK1	BioLegend
CD45RO	BV510	UCHL1	BioLegend
CD137	PE	4B4-1	BioLegend
CCR7	APC	G043H7	BioLegend
